# The Tripartite Nexus: Autophagy, Cancer, and Tripartite Motif-Containing Protein Family Members

**DOI:** 10.3389/fphar.2020.00308

**Published:** 2020-03-11

**Authors:** Michael A. Mandell, Bhaskar Saha, Todd A. Thompson

**Affiliations:** ^1^ Department of Molecular Genetics and Microbiology, University of New Mexico Health Sciences Center, Albuquerque, NM, United States; ^2^ Autophagy, Inflammation and Metabolism Center of Biomedical Research Excellence, University of New Mexico Health Sciences Center, Albuquerque, NM, United States; ^3^ Department of Pharmaceutical Sciences, University of New Mexico College of Pharmacy, Albuquerque, NM, United States

**Keywords:** autophagy, cancer therapy, tripartite motif (TRIM) family, Sequestosome 1 (p62/SQSTM1), selective autophagy cargo receptor, autophagy regulation, cancer, autophagy modulating drugs

## Abstract

Autophagy is a cellular degradative process that has multiple important actions in cancer. Autophagy modulation is under consideration as a promising new approach to cancer therapy. However, complete autophagy dysregulation is likely to have substantial undesirable side effects. Thus, more targeted approaches to autophagy modulation may prove clinically beneficial. One potential avenue to achieving this goal is to focus on the actions of tripartite motif-containing protein family members (TRIMs). TRIMs have key roles in an array of cellular processes, and their dysregulation has been extensively linked to cancer risk and prognosis. As detailed here, emerging data shows that TRIMs can play important yet context-dependent roles in controlling autophagy and in the selective targeting of autophagic substrates. This review covers how the autophagy-related actions of TRIM proteins contribute to cancer and the possibility of targeting TRIM-directed autophagy in cancer therapy.

## Introduction

Macroautophagy is a promising new target for cancer treatment as this cellular pathway has both cancer-suppressing and cancer-promoting mechanisms. Macroautophagy (autophagy hereafter) is a process of cellular self-digestion that involves the sequestration of cytoplasmic contents into a vesicle (the autophagosome) that fuses with the lysosome where it is degraded. The “core” molecular machinery that is required for autophagy consists of more than 30 proteins. These proteins were mostly identified in yeast and their functions are conserved in human cells (Klionsky et al., 2011). However, as the physiological roles of autophagy have been expanded in higher organisms, the number of proteins involved in mammalian autophagy is increased relative to what is seen in single-celled organisms. Autophagy has been classified as being either “bulk” or “selective”, the latter indicating the ability of the autophagy machinery to identify and selectively degrade substrates. Selective autophagy is further classified into “–phagies”, denoting the particular substrates degraded: for example, mitophagy is the autophagic degradation of mitochondria, ERphagy involves autophagy of endoplasmic reticulum, ferritinophagy refers to autophagic degradation of ferritin, and so forth. While the different “phagies” all require the same core autophagy machinery, they can vary in terms of their upstream regulators and in the factors required for specific cargo identification (Kirkin and Rogov, 2019). This variability in mechanism opens the possibility to the selective pharmacological modulation of certain autophagic activities.

Autophagic degradation of cytoplasmic contents can generate molecules for biosynthesis or energy during times of cellular starvation. Additionally, autophagy plays an important cytoplasmic quality control function that can eliminate specific proteins, toxic protein aggregates, unnecessary or non-functional organelles, and intracellular pathogens from cells. These pro-survival functions of autophagy have been of interest as potential targets of cancer therapy (Levy et al., 2017), and work in experimental rodent cancer models has demonstrated that a variety of tumors require functional autophagy in the tumor cells themselves and in healthy tissue (Poillet-Perez and White, 2019). Furthermore, there are indications that autophagy can render cancer cells more resistant to chemotherapy (Sui et al., 2013; Pan et al., 2019). Together, these findings strongly indicate that pharmacological inhibition of autophagy may be a promising approach to cancer therapy. Nevertheless, there is a problem: complete inhibition of autophagy by inducible whole-body knockout of the core autophagy factor ATG7 is lethal in mice (Karsli-Uzunbas et al., 2014), suggesting the strong likelihood of unacceptably severe side effects if autophagy inhibition were to be tried in human cancer patients. A more tractable approach to modulate autophagy in cancer may be to target specific components of the varieties of autophagic processes.

The challenge of targeting specific elements of autophagy could potentially be alleviated if there was some way to specifically target a cancer-promoting phagy while allowing other varieties of autophagy to proceed normally. Conceptually, this would be done by targeting proteins with phagy-specific functions rather than by targeting the core machinery or lysosomal function to block all autophagy. One possible way of doing this is through the tripartite motif family of proteins (TRIMs). This large protein family has emerged as possessing a wide variety of actions on autophagy regulation and action. Importantly, many TRIMs have very strong connections to oncogenesis or cancer progression. The purpose of this review is to detail how TRIMs intersect with and orchestrate autophagy and to discuss how TRIM-mediated autophagy may affect oncogenesis, cancer progression, and cancer therapy.

### The TRIM Family

**Figure 1:**
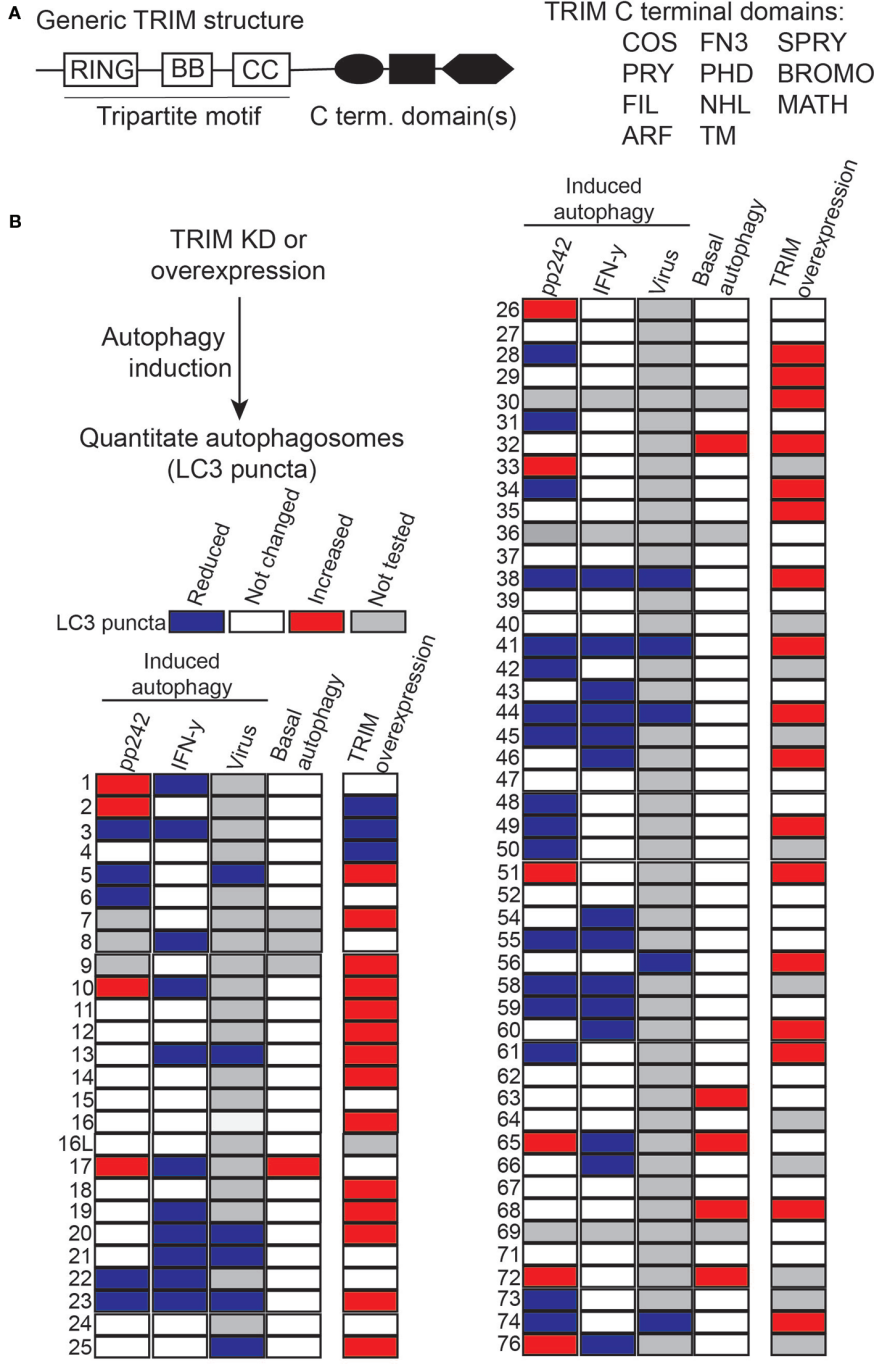
Many tripartite motif-containing protein family members (TRIMs) act as autophagy regulators. **(A)** Left, schematic of generic TRIM protein domain organization. Typical TRIMs have N-terminal RING domains (RING), one or two B box domains (BB), a coiled-coil (CC) domain and may have one or more C terminal domains. Right, list of C terminal domains present in TRIM family proteins. **(B)** The results of several previously published TRIM siRNA or over-expression screens are summarized here in heat map format. In all experiments, cells were transfected with TRIM siRNA or expression plasmids and treated or not with a known inducer of autophagy (e.g. pp242) prior to imaging-based quantitation of cytoplasmic LC3B or GFP-LC3B puncta (autophagosomes). TRIMs that significantly increased or decreased autophagosome abundance relative to negative controls are colored red or blue, respectively. Changes in the abundance of autophagosomes can result from either increased autophagy activation or decreased autophagy flux, thus in isolation these data do not indicate mechanisms of individual TRIMs on autophagy but illustrate the broad involvement of TRIMS in autophagy regulation.

The TRIM family of proteins is structurally distinguished by having a cluster of domains starting with an N-terminal RING domain, followed by one or two B box domains, and then a coiled-coil domain (CCD; **Figure 1A**) (Reymond et al., 2001). The RING domain confers upon TRIMs their catalytic function as E3 ligases, and individual TRIMs have been shown to directly ubiquitylate, SUMOylate, or NEDDylate themselves and/or their interacting partners (Ivanov et al., 2007; Noguchi et al., 2011; Fletcher et al., 2015). While the overwhelming majority of TRIMs possess a RING domain, there are some exceptions (e.g. TRIM16, TRIM20). Interestingly, TRIM16 still has ubiquitin ligase activity due to a cryptic RING-like fold in its B box domain (Bell et al., 2012), emphasizing that the enzymatic activity of TRIMs should be determined empirically. The B box and CCD both mediate protein-protein interactions, with the CCD allowing for TRIM hetero- and homodimerization. At their C terminus, most TRIMs have one or more additional domains with the SPRY domain being the most common variant in human TRIMs. The SPRY domain is important for mediating protein-protein interactions such as the interaction between TRIM5 and retroviral capsids, while other C terminal domains have different interacting specificities (e.g. PHD domain can bind to chromatin) or even have enzymatic activities (the ADP-ribosylation factor/ARF domain of TRIM23).

TRIMs are a metazoan-specific protein family, with seven TRIMs found in the genome the fruit fly *Drosophila melanogaster* and 18 TRIMs in the *Caenorhabditis elegans* genome (Sardiello et al., 2008). The number of TRIM genes is substantially elevated in vertebrates, with more than 200 TRIMs or TRIM-like genes found in the zebrafish (*Danio rerio*) genome (Sardiello et al., 2008). The human genome includes more than 80 TRIMs which have been assigned into eleven sub-families based on their domain organization (Short and Cox, 2006; Ozato et al., 2008). Many of these genes encode for multiple isoforms, thus further expanding the protein sequence diversity and possibly the functionality of TRIM proteins. At a cellular level, these functions include governing gene expression, regulating signal transduction pathways, contributing to cytoplasmic quality control, direct antiviral action, and effects on cell survival and metabolism. At the organismal level, TRIMs play important roles in development and in immune regulation, and alterations in TRIM protein function/expression are linked to a variety of diseases including cancer (Watanabe and Hatakeyama, 2017; Park et al., 2020).

### Alterations in TRIM Expression Is a Hallmark of Many Cancers

Many TRIM proteins are found as relevant biomarkers of cancer, where they may show decreased or increased levels of expression (**Table 1**). A significant decrease in TRIM expression associated with cancers is suggestive of a tumor suppressive role. In contrast, a significant overexpression of TRIM proteins may reflect a contribution to cancer development and/or cancer progression. TRIMs with the greatest association with cancer include 11, 14, 24, 25, 27, 28, 29, 33, 37, 44, and 59, each associated with at least five different cancers. It is likely the expression of TRIMs in cancers is relevant to the development and/or progression of the disease and TRIM expression may have prognostic value for cancer. Furthermore, TRIMs associated with specific cancers may provide insight into the development of novel TRIM targeted cancer therapies. Importantly, associations between individual TRIMs and different cancers are regularly being discovered, strongly predicting that the list presented in **Table 1** will grow with further study. The following paragraphs details a few of the connections between TRIMs and individual cancers.

**Table 1:** TRIM expression changes found in cancers.

TRIM (Alias)	Cancer(s)	Reference(s)
2*	Colorectal; clear cell renal cell↓; osteosarcoma	Cao et al. (2019); Xiao et al. (2018); Qin et al. (2018)
3* (BERP)	Gastric↓; Liver↓	Fu et al. (2018); Chao et al. (2014)
8* (GERP)	Glioma↓; laryngeal↓	Micale et al. (2015); Carinci et al. (2007)
11*	Breast; glioma; liver; lung; ovarian	Song W. et al. (2019); Di et al. (2013); Chen et al., (2017b); Liu J. et al. (2017); Zhang et al. (2017); Huang et al. (2019); Wang X. et al. (2016); Chen et al. (2017a)
13*	Breast↓; lung↓; multiple myeloma	Chen et al. (2019); Xu L. et al. (2019); Gatt et al. (2013)
14*	Gastric; glioma; glioblastoma; liver; tongue squamous cell carcinoma	Wang F. et al. (2018); Tan Z. et al. (2018); Feng et al. (2019); Dong and Zhang (2018); Su et al. (2016)
15	Gastric↓	Chen W. et al. (2018)
16* (EBBP)	Breast↓; liver↓; lung↓; melanoma↓; prostate↓	Yao et al. (2016); Li et al. (2016); Huo et al. (2015); Sutton et al. (2014); Qi L. et al. (2016)
21* (Ro52)	B-cell lymphoma; breast↓; liver↓	Brauner et al. (2015); Zhou et al. (2018); Ding et al. (2015)
22*	Lung; Wilm's tumor↓	Liu et al. (2017b); Zirn et al. (2006)
24 (TIF1α)	Bladder; breast; cervical; colorectal; esophageal↓; gastric; glioblastoma; glioma; head & neck; liver; lung; nasopharyngeal; prostate	Xue D. et al. (2015); Tsai et al. (2010); Lin et al. (2017); Wang et al. (2017); Chi et al. (2016); Miao et al. (2015); Lv et al. (2017); Zhang et al. (2015); Cui et al. (2013); Zhu et al. (2018); Li et al. (2012); Wang P. et al. (2018); Groner et al. (2016); Offermann et al. (2019)
25* (EFP)	Breast; colorectal; endometrial↓; lung; ovarian; prostate	Suzuki (2005); Ivanov et al. (2007); Dai et al. (2010); Qin et al. (2016); Sakuma et al. (2005); Takayama et al. (2018)
26*	Liver↓	Wang et al. (2015b)
27* (RFP)	Breast; colorectal; endometrial; lung; ovarian	Tezel et al. (2009); Zhang Y. et al. (2018); Tsukamoto et al. (2009); Iwakoshi et al. (2012); Ma et al. (2016)
28* (TIF1β; KAP1)	B-cell non-Hodgkin lymphoma; breast; colorectal; gastric; glioma; liver; lung; ovarian; pancreatic; prostate; thyroid; Wilm's tumor	Zhang P.-P. et al. (2018); Ho et al. (2009); Fitzgerald et al. (2013); Yokoe et al. (2010); Wang et al. (2013); Qi Z.-X. et al. (2016); Wang Y. et al. (2016); Liu et al. (2017a); Cui et al. (2015); Yu et al. (2014); Fong et al. (2018); Martins et al. (2013); Halliday et al. (2018)
29* (ATDC)	Bladder; cervical; colorectal; esophageal; liver↓; lung; nasopharyngeal; oral↓; osteosarcoma; pancreatic; prostate↓	Palmbos et al. (2015); Tan et al. (2016); Xu R. et al. (2016); Jiang et al. (2013); Xu W. et al. (2016); Lai et al. (2013); Xu M. et al. (2019); Song et al. (2015); Chen et al. (2016); Zhou et al. (2016); Harris et al. (2015); Zeng et al. (2017); Sun et al. (2014); Kanno et al. (2014)
31*	Liver; pancreatic	Guo et al. (2018); Yu et al. (2018)
32* (HT2A)	Breast; gastric; liver; lung	Zhao et al. (2018); Ito et al. (2017); Wang C. et al. (2018); Cui et al. (2016); Yin et al. (2019)
33* (TIF1γ)	Breast; colorectal; glioblastoma↓; liver↓; renal↓	Kassem et al. (2015); Xue J .et al. (2015); Ding et al. (2014); Jingushi et al. (2015)
35*	Liver	Jia et al. (2011)
36	Prostate↓	Fujimura et al. (2014); Liang et al. (2018); Kimura et al. (2018)
37*	Breast; colorectal; esophageal; gastric; glioma; liver; lung; osteosarcoma	Bhatnagar et al. (2014); Tuna et al. (2012); Hu and Gan (2017); Wu et al. (2018); Chen D. et al. (2018); Tang et al. (2018); Jiang et al. (2015); Dong et al. (2018); Li et al. (2018); Tao et al. (2017)
44*	Breast; cervical; esophageal; gastric; lung; melanoma; ovarian; testicular	Kawabata et al. (2017); Liu et al. (2019); Peters et al. (2010); Ong et al. (2013); Kawaguchi et al. (2017); Kashimoto et al. (2012); Xing et al. (2016); Wei et al. (2019); Liu S. et al. (2018); Yamada et al. (2017)
47	Breast; colorectal; lung; prostate	Wang et al. (2020); Liang et al. (2019); Han et al. (2017); Fujimura et al. (2016)
50*	Ovarian↓	Qiu et al. (2019)
58*	Lung↓	Diaz-Lagares et al. (2016)
59* (IFT80)	Breast; cervical; colorectal; gastric; lung; osteosarcoma; ovarian	Liu Y. et al. (2018); Tan P. et al. (2018); Aierken et al. (2017); Wu et al. (2017); Zhou et al. (2014); Hao et al. (2017); Liang et al. (2016); Wang Y. et al. (2018); Zhang et al. (2019)
62 (DEAR1)	Acute myeloid leukemia↓; breast↓; lung↓	Quintás-Cardama et al. (2015); Lott et al. (2009); Quintás-Cardama et al. (2014)
63* (MuRF1)	Breast	Li et al. (2019)
65*	Bladder; liver; lung	Wei et al. (2018); Yang Y.-F. et al. (2017); Wang X.L. et al. (2016a)
66* (TIF1δ)	Osteosarcoma	Chen et al. (2015)
68*	Prostate	Miyajima et al. (2008)
72* (MG53)	Colorectal↓	Chen Z. et al. (2018); Fernández-Aceñero et al. (2019)
L2 (SPRYD6)	Oral; triple-negative breast cancer	Hayashi et al. (2019); Song X. et al. (2019)

**Figure 2:**
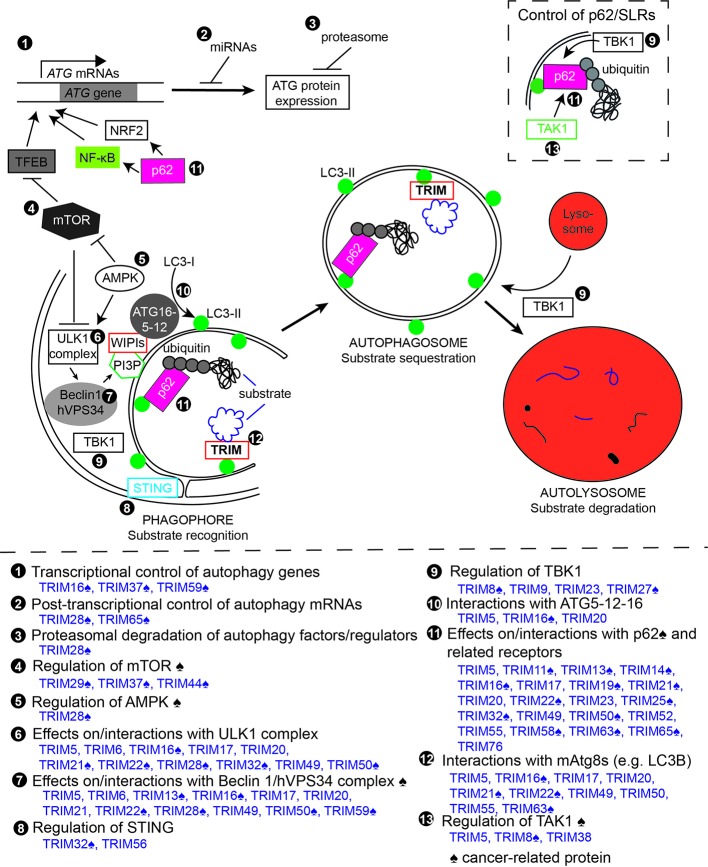
Tripartite motif-containing protein family members (TRIMs) regulate the autophagy pathway at multiple points. Top, schematic of different steps/stages of the autophagy pathway. Circled numbers indicate steps of the autophagy pathway or autophagy regulators and factors that are impacted by individual TRIMs. Bottom, summary of TRIM actions in autophagy. Circled numbers correspond with those on the schematic. ♠ symbol indicates proteins with reported cancer relevance.

*Autophagy associated TRIMs per **Figures 1** and **2**.

↓ indicates the TRIM protein was found to be decreased in expression compared to normal tissue. No arrow indicates the TRIM protein was overexpressed in the corresponding cancer compared to normal tissue.

#### Lung Cancer

Lung cancer has the highest mortality among cancers in the U.S. (Siegel et al., 2020), in part due to the poor response of current cancer chemotherapeutic regimens for lung cancer. In addition to the importance of identifying new lung cancer biomarkers, the identification of novel therapeutic targets or approaches to increase the efficacy of lung cancer chemotherapies is critically needed. Many TRIMs are altered in expression in lung cancer (**Table 1**). Of these, TRIMs 13, 16, 58, and 62 have reduced levels of expression, whereas the majority of TRIMs associated with lung cancer are increased in expression. TRIM13 expression is reduced in non-small-cell lung cancer (NSCLC), where its overexpression was found to inactivate NF-κB (Xu L. et al., 2019). TRIM16 was also found decreased in NSCLC with concurrent upregulation of the sonic hedgehog pathway, suggesting a role for TRIM16 in epithelial-mesenchymal-transition in NSCLC (Huo et al., 2015). Interestingly, hypermethylation of TRIM58 in lung cancer may account for its down-regulation (Diaz-Lagares et al., 2016). Overexpression of TRIMs 11, 22, 44, 47, 59, and 65 were correlated with poor prognosis in lung cancers (Liu et al., 2013; Wang X.-L. et al., 2016; Xing et al., 2016; Han et al., 2017; Hao et al., 2017; Liu et al., 2017b; Huang et al., 2019; Yin et al., 2019). Overexpression of TRIM44 induced mTOR signaling, epithelial-mesenchymal transition, and cyclin/CDK upregulation in lung cancer cells (Xing et al., 2016). Poor prognosis of lung cancer patients was observed in those overexpressing TRIM27 (RFP) and possessing epidermal growth factor receptor mutations (Iwakoshi et al., 2012). The multitude of TRIMs found increased in lung cancers may serve as promising targets for improved lung cancer therapy.

#### Breast Cancer

As listed in **Table 1**, breast cancer has been associated with at least 15 different TRIM proteins. Decreased levels of TRIM expression in breast cancer were observed with TRIMs 13, 21, and 62. Chen et al. (2019) found that decreased TRIM13 expression was associated with worse distant metastasis free survival, disease specific survival, metastatic relapse free survival, and relapse free survival. Zhou et al. (2018) found decreased TRIM21 expression correlated with poor overall survival in breast cancer patients. Reduced TRIM62 (DEAR1) expression was found in many breast cancer tissues and was found to strongly correlate with early-onset breast cancer (Lott et al., 2009). In a 3D cell culture model, restoration of TRIM62 expression inhibited uncontrolled cell growth and directed the cells to form organoids reminiscent of health breast tissue (Lott et al., 2009). In contrast, TRIM11 levels were found increased in breast cancer tissues, where TRIM11 may act through the AKT/GLUT1 signaling pathway in breast cancer (Song W. et al., 2019). Elevated TRIM24 and TRIM37 in breast cancer may act through modifications of histone proteins, H2A and H3, respectively (Tsai et al., 2010; Bhatnagar et al., 2014). Elevated levels of TRIM32 and TRIM44 were associated with actions on NF-κB pathways in breast cancer (Kawabata et al., 2017; Zhao et al., 2018). Interestingly, TRIM59 was found upregulated in metastatic breast cancer, where it was observed to suppress the selective autophagic degradation of a tumor suppressor (Tan P. et al., 2018), underscoring an autophagic role for TRIM proteins in cancer.

#### Liver Cancer

Though the incidence of liver cancer is relatively low in the U.S., the mortality rate for this cancer is high (Siegel et al., 2020). TRIMs 3, 16, 21, and 29 were found at reduced levels in liver cancers (**Table 1**), where this reduced expression was consistently found associated with poorer prognosis among liver cancer patients. In contrast, TRIMs 11, 14, 24, 28, 31, 32, 37, and 65 have been found elevated in human liver cancers.

#### Colorectal Cancer

Colorectal cancer is the third most common cancer in both incidence and mortality among men and women in the U.S. (Siegel et al., 2020). TRIM72 expression is reduced in the serum of colon cancer patients and in colon cancer tumors (Chen Z. et al., 2018; Fernández-Aceñero et al., 2019). In contrast, most TRIMs associated with colorectal cancers have been found up-regulated, including TRIMs 2, 24, 25, 27, 29, 33, 37, 47, and 59 (**Table 1**). Up-regulation of TRIM47 in colorectal cancers was associated with SMAD4 degradation, enhancing growth and invasion of colorectal cancer cells (Liang et al., 2019).

#### Prostate Cancer

The androgen receptor possesses a key function in prostate cancer progression serving as the main target for the treatment of advanced, hormone-responsive disease (Yuan et al., 2014). TRIM36 expression is increased in response to androgen and has a prostate cancer suppressive role that includes inhibiting prostate cancer cell proliferation and migration while promoting prostate cancer cell death (Kimura et al., 2018; Liang et al., 2018). Reduced levels of TRIM36 are associated with advanced stages of prostate cancer (Fujimura et al., 2014; Kimura et al., 2018; Liang et al., 2018) and TRIM36 was reported to be an independent predictor of survival in prostate cancer patients (Kimura et al., 2018). Like TRIM36, expression TRIMs 16 and 29 is decreased in prostate cancer, suggesting that these proteins may act as tumor suppressors in normal prostate tissue. In contrast, high expression levels of TRIMs 24 and 28 are associated with more advanced prostate cancer disease, particularly in androgen non-responsive, castration resistant cancer. TRIM24 can augment androgen receptor signaling (Groner et al., 2016), apparently downstream of the actions of TRIM28 (Fong et al., 2018). Increased levels of TRIMs 25, 47, and 68 are also associated with poorer prognosis of prostate cancer (**Table 1**).

### TRIMs Impact Cancer Through Multiple Mechanisms

#### Chromosomal Translocations Involving TRIMs That Result in Oncogenic Gain-Of-Function

A number of TRIM genes are associated with chromosomal translocations that likely contribute to oncogenesis. One of the most investigated involves a translocation between the *TRIM19* gene (also known as *PML*) on chromosome 15 and the retinoic acid receptor alpha (RARα) gene located on chromosome 17, which is associated with acute promyelocytic leukemia (Cambiaghi et al., 2012). This fusion protein acts by repressing genes associated with retinoic acid signaling. The RET gene on chromosome 10 has been found in translocations with a number of TRIM genes including TRIM24, TRIM27, and TRIM33 associated with papillary thyroid cancer (Klugbauer and Rabes, 1999), lymphoma (Takahashi et al., 1985), and non-small cell lung carcinoma (Lin et al., 2015), respectively. Similarly, the BRAF gene has been found translocated with TRIM4 in lung cancer (Shim et al., 2015) and TRIM24 in both melanoma (Hutchinson et al., 2013) and lung cancer (Nakaoku et al., 2014) and the FGFR1 gene is translocated with TRIM24 in myeloproliferative syndrome (Belloni et al., 2005). These fusion proteins lead to the unregulated activity of the RET, BRAF, or FGFR1 kinases, resulting in the activation of multiple pro-survival signaling pathways. In summary, a number of TRIM-containing oncogenic gain-of-function fusion genes have been found with profound effects in oncogenesis.

#### Contribution of TRIMs to Cancer “Stemness”

A small sub-population of cancer cells have properties reminiscent of embryonic stem cells including a high degree of cellular plasticity, resistance to cell death, and the capacity of self-renewal. These cancer stem cells are important contributors to metastasis, drug resistance, and cancer recurrence, leading to an interest in targeting them as part of cancer therapy (Yang et al., 2020). Several TRIMs can regulate pathways seminal to cancer stemness including STAT signaling, AKT signaling, NANOG-Sox2-Oct-3/4 networks, and pathways related to epithelial-mesenchymal transition (EMT). In this section we cite a few examples of how TRIMs can regulate stemness, a topic that was recently reviewed by Jaworska et al. (2020). TRIM28 is reported to maintain of Oct-3/4-Sox2-NANOG expression in breast cancer cells (Czerwinska et al., 2017). TRIM24 is also reported to promote cancer stemness in glioblastoma by enhancing STAT3-mediate transcriptional activation (Lv et al., 2017). Additionally, TRIMs 14 and 24 have been shown to enhance EMT through promoting AKT signaling in gastric and colorectal cancer, respectively (Wang F. et al., 2018; Zhang Y. et al., 2018). In contrast to TRIMs that up-regulate stemness pathways, TRIM16 has been associated as a negative regulator of stemness in breast and ovarian cancer cells (Yao et al., 2016; Tan et al., 2017). In both cases, TRIM16 acted to reduce expression of the Hh signaling-activated transcription factor Gli-1, a positive regulator of cancer stem cell self-renewal. Importantly, autophagy is another key contributor to cancer stem cells promoting their longevity as quiescent cells (Vera-Ramirez et al., 2018) and its downregulation is associated with cancer stem cell reactivation (La Belle Flynn et al., 2019). TRIMs are major regulators of autophagic processes, and their autophagic actions may constitute another TRIM-dependent contribution to cancer stemness.

#### Modulation of p53 Stability and Activity by TRIMs

The tumor suppressor protein p53 promotes genomic stability and can induce cell cycle arrest and apoptosis resulting from extensive cellular DNA damage. Interactions between TRIM proteins and p53 are well-established and have recently been reviewed in detail (Valletti et al., 2019). TRIMs 11, 21, 24, 25, 28, 29, 31, 32, 39, and 59 all negatively regulate p53. Mechanistically, TRIMs can directly ubiquitylate p53, leading to its consequent degradation or sequestration in the cytoplasm where it cannot impact gene expression or carry out its pro-apoptotic or cell-cycle controlling functions.

In addition to being subject to proteasomal degradation, multiple studies have demonstrated that p53 can also be regulated by delivery to the lysosome for degradation by the autophagy pathway (White, 2016). Autophagy regulation is a very prominent feature of many TRIMs. Collectively, recent studies have identified individual TRIMs that impact stages of the autophagy pathway. The following sections will focus on the mechanisms whereby TRIMs impact autophagy and highlight examples of how TRIM-directed autophagy contributes to cancer.

### Regulation of Autophagy by TRIMs

In addition to these activities in cancer, TRIMs also have been shown to impact oncogenesis and tumor progression through their actions on autophagy. A panel of published siRNA screens have demonstrated that a surprisingly high percentage of human TRIMs appear to regulate autophagy in cells under basal autophagy conditions (Mandell et al., 2014) or in response to autophagy induction by mTOR inhibition (Mandell et al., 2014; Sparrer et al., 2017), interferon γ stimulation (Kimura et al., 2015), lysosomal damage (Chauhan et al., 2016), or viral infection (Sparrer et al., 2017). Collectively, these screens identified 49 TRIMs whose knockdown either decreased or increased the number of autophagosomes in cells (**Figure 1B**). Additional sets of TRIMs have been identified as having autophagy regulatory roles under other experimental conditions. The fact that so many TRIMs were identified in these screens illustrated that some of the actions of TRIMs in autophagy are context dependent with some TRIMs contributing to defined subsets of autophagic outputs. For example, TRIM16 was uniquely required among TRIMs for an autophagic response to lysosomal damage but was dispensable for autophagy induced by mTOR inhibition, interferon γ, or viruses. The context-specific action of TRIMs in autophagy is important because it suggests that the modulation of a specific TRIM may affect only the subset of autophagic activities governed by that TRIM. The ability to precisely alter the cancer-related subset of autophagic activities may be therapeutically beneficial. Additionally, the broad requirement for TRIMs in autophagy suggested that they may act *via* non-redundant mechanisms. This notion is supported by subsequent studies, which have demonstrated that some TRIMs affect the cellular abundance of autophagy-related proteins whereas other TRIMs appear to affect the activation status of autophagy regulators and/or alter their protein-protein interactions (**Figure 2**).

#### TRIMs Regulate Autophagy at the mRNA Level

Several TRIMs have been shown to affect the transcription of autophagy genes. In some cases, this is through TRIM actions on transcription factors that activate expression of autophagy-related genes. For example, the expression of TRIM59 in the lung carcinoma cell line H1299 inhibits autophagy by negatively regulating the expression of *Becn1* mRNA (Han et al., 2018), an effect that was connected to TRIM59's observed inhibitory action on NF-κB activation. TRIM37, a known oncogene (Bhatnagar et al., 2014), suppresses autophagic flux and inhibits the activation and nuclear translocation of the pro-autophagy transcription factor TFEB (Wang W. et al., 2018). Conversely, TRIM16 promotes its own expression along with that of the autophagy receptor p62 by driving Nrf2 activation under conditions of oxidative stress (Jena et al., 2018). TRIM16 is also found in protein complexes with TFEB (Chauhan et al., 2016), but how this interaction shapes TFEB activation separately from the role of TRIM16 in maintaining lysosomal health has not been fully explored. It is likely that additional TRIMs will be identified that can regulate autophagy by effecting diverse signal transduction pathways that result in the activation of transcription factors (e.g. IRF3, AP1, Nf-κB).

In addition to regulating transcription factor activity, some TRIMs localize to the nucleus and can directly act as transcriptional regulators or co-regulators. While a subset of TRIMs have a C terminal domain (plant homeodomain, PHD) that mediates chromatin binding, some TRIMs lacking these domains can localize to the nucleus and affect gene expression: an example being TRIM22 which was shown to reduce retroviral gene expression (Kajaste-Rudnitski et al., 2011). The transcriptional regulatory activities of a TRIM on autophagy was first demonstrated for the PHD domain-containing TRIM28 (also known as KAP1, **Table 1**) (Barde et al., 2013). Hematopoietic-specific knockout of TRIM28 resulted in abnormal erythroblasts that contained elevated numbers of mitochondria. Accordingly, TRIM28 knockout erythroblasts expressed substantially lower levels of mRNAs coding for core autophagy factors (e.g. *Ulk1*, *Becn1*, *Atg12*) and for proteins with mitophagy-specific functions (*Nix* and *Bnip3L*). Mechanistically, TRIM28 was found to repress the expression of miRNAs that target autophagy factors. Similarly, TRIM65 promotes autophagy by preventing miRNA-based down-regulation of ATG7 in a non-small-cell lung cancer cell line (Pan et al., 2019). In this study, the TRIM65 knockdown potentiated the cytotoxic effects of cisplatin.

#### Actions of TRIMs on Autophagy-Regulating Signaling Pathways

The autophagy pathway is central to many cellular functions under both homeostatic and stress conditions, and hence a large number of signal transduction pathways have roles in positively or negatively regulating autophagy. The mTOR and AMPK signaling pathways, both of which are involved in sensing a cell's nutritional status and are of critical importance to cancer, are the best-known autophagy regulating pathways. mTOR (mammalian target of rapamycin) is activated under amino acid replete conditions and in turn activates signaling that promotes anabolic processes and cellular growth. Active mTOR attenuates the expression of autophagy genes by inhibiting the activation and nuclear localization of TFEB and other MiTF transcription factors through direct phosphorylation. mTOR also directly inhibits the activation of autophagosome initiation by phosphorylation of the most upstream autophagy factor ULK1 at serine 757. AMPK (5' AMP-activated protein kinase) is activated by low glucose conditions in cells and directly opposes the actions of mTOR. When activated, AMPK phosphorylates mTOR, leading to the disassembly and inactivation of mTOR complexes. AMPK also phosphorylates ULK1 in an activating manner at Ser317, Ser555, and Ser777, resulting in autophagy activation (Galluzzi et al., 2014).

Several TRIMs are now known to regulate autophagy through actions on mTOR or AMPK signaling. For instance, TRIM37 (**Table 1**) was recently reported to physically interact with mTOR complex components and to promote the assembly of active mTOR complexes at the lysosome (Wang W. et al., 2018). TRIM37-deficient cells carryout unregulated autophagy. Interestingly, TRIM37-deficient cells become “autophagy addicted”, and so inhibition of autophagy flux in these cells leads to pronounced cell death (Wang W. et al., 2018). TRIM29 and TRIM44 (**Table 1**) are also reported to affect mTOR signaling (Xing et al., 2016; Zhou et al., 2016).

The ability of AMPK to induce autophagy is also subject to regulation by TRIMs. In a subset of cancers, TRIM28 ubiquitylates the AMPKα1 subunit, resulting in its proteasomal degradation and repression of autophagy (Pineda et al., 2015). This effect is mediated by two proteins whose expression is largely cancer-specific, the melanoma antigen A3 and A6 (MAGE-A3/6), which interact with TRIM28 and recruit it to AMPKα1. AMPK activity has also been linked to the pro-cancer kinase TAK1 (Xie et al., 2006; Herrero-Martin et al., 2009), and TAK1 is in turn activated by TRIMs 5 (Pertel et al., 2011) and 8 (Li et al., 2011) and inhibited by TRIM38 (Hu et al., 2014), likely through autophagic degradation of TAK1 complex components.

The STING-TBK1 signaling axis is another autophagy-regulating pathway whose activity is orchestrated by multiple TRIMs. STING is a crucial component of cytosolic DNA signaling pathways. Under homeostatic conditions, STING, which contains four transmembrane domains, is localized to the endoplasmic reticulum membrane in an inactive state. In response to cytosolic DNA detection, STING undergoes a conformational change that allows for the recruitment of the kinase TBK1. The STING-TBK1 complex re-localizes to the ER-Golgi intermediate compartment, where TBK1 can phosphorylate and activate the transcription factor IRF3 (Chen Q. et al., 2016). TBK1 can also be activated by other pattern recognition receptors (Louis et al., 2018). These two proteins have key roles in autophagy regulation. STING has been shown to be important for autophagy induction in response to various microbial stimuli (Watson et al., 2015; Moretti et al., 2017), and cytosolic DNA-activated STING was recently shown to provide a membrane source for autophagosome formation independently of the “core” autophagy upstream regulator ULK1 and the hVPS34/Beclin 1 complex (Gui et al., 2019). TBK1 also plays important roles in autophagy regulation, at least some of which are independent of STING. For instance, TBK1-mediated phosphorylation of syntaxin 17 is required for the earliest steps of autophagosome formation (Kumar et al., 2019) and TBK1 also has a role in allowing for autophagic maturation (Pilli et al., 2012). These activities may be in addition to or in conjunction with TBK1's roles in promoting autophagic cargo selectivity through actions on autophagy receptors p62, NDP52, and optineurin (Pilli et al., 2012; Richter et al., 2016; Vargas et al., 2019). Both TRIM56 and TRIM32 have been shown to potentiate the STING-TBK1 pathway by carrying out the K63-linked poly-ubiquitylation of STING (Tsuchida et al., 2010; Zhang et al., 2012), while the mouse-specific TRIM30α catalyzes K48-linked ubiquitylation of STING, resulting in its degradation (Wang et al., 2015a). TRIM27 promotes the proteasomal degradation of TBK1 (Zheng et al., 2015), while other TRIMs can affect the activity of TBK1 by modifying its protein-protein interactions (Qin et al., 2016; Ye et al., 2017). Whereas autophagy was not addressed in the studies cited above, TRIM23 has been demonstrated to affect virus-induced autophagy through direct actions on TBK1 (Sparrer et al., 2017). TRIM23 is unique among the human TRIM family in that it is the only TRIM to feature an ADP ribosylation factor-like ARF domain as a C terminal domain. This domain is required for the interaction between TRIM23 and TBK1 and for TRIM23-mediated autophagy. Both genetic and pharmacological inhibition of TBK1 impaired autophagy driven by TRIM23 expression. This finding provided the first evidence that a TRIM could regulate autophagy by acting on TBK1, suggesting the possibility that other TRIMs may act similarly.

#### Actions of TRIMs on Autophagy Machinery

The previous sections dealt with mechanisms whereby TRIMs regulate autophagy indirectly at the transcriptional level or by affecting upstream signal transduction pathways that affect autophagy. In this section, we discuss evidence that multiple TRIMs directly interact with and modulate the activity of the conserved core autophagy machinery. The first indication that TRIMs as a family could directly intersect with the autophagy machinery was published in 2014, when TRIM5, TRIM6, TRIM17, TRIM22, and TRIM49 were shown to interact with the autophagy regulators ULK1 and Beclin 1 (Mandell et al., 2014). ULK1 (the mammalian homologue of yeast Atg1) is the most upstream autophagy regulator. One of the roles of ULK1 in autophagy is to activate the Beclin 1/hVPS34 complex, which it does through phosphorylation of Beclin 1 (Russell et al., 2013). This process was potentially enhanced by expression of the TRIMs listed above, which recruited ULK1 into Beclin 1 multi-molecular complexes (Mandell et al., 2014) in co-immunoprecipitation experiments. Further studies with TRIM5 showed that it also interacted with ATG14L1 and AMBRA1, both proteins that interact with the Beclin 1 complex. Additional studies have broadened the list of TRIMs that interact with the ULK1 and/or Beclin 1 complexes to include TRIMs 13, 16, 20, 21, 28, 32, and 50 (Yang et al., 2013; Kimura et al., 2015; Chauhan et al., 2016; Fusco et al., 2018; Di Rienzo et al., 2019; Ji et al., 2019). However, the finding that a TRIM interacts with these upstream regulators of autophagy does not necessarily prove that it acts to promote autophagy, as exemplified by TRIM17 which binds to ULK1 and Beclin 1 yet was found to inhibit autophagosome formation (Mandell et al., 2014; Mandell et al., 2016). Instead, TRIM17 promoted the formation of inhibitory Beclin 1 complexes including the protein Mcl-1 (Mandell et al., 2016).

Active Beclin 1 complexes generate phosphatidylinositol 3-phosphate (PI3P) at autophagosome initiation sites. PI3P recruits proteins including ATG16L1 and ATG5 that carry out the elongation of the autophagosome membrane. A key part of this process involves the lipidation of the mammalian Atg8 orthologues (LC3 and GABARAP proteins; mAtg8s). Lipidated mAtg8 proteins are important for the elongation of the autophagosome membrane, the closure of the autophagosome, and its fusion with lysosomes. TRIM5, TRIM16, and TRIM20 have all been reported to form protein complexes with ATG16L1 and TRIM5 was also shown to co-immunoprecipitate with ATG5 (Kimura et al., 2015; Ribeiro et al., 2016). Whether these interactions specifically modulate mAtg8 lipidation and autophagosome membrane elongation has not yet been demonstrated.

One critical question is whether the enzymatic activity of TRIMs as E3 ligases is important for their actions in autophagy. The answer to this question appears to be “sometimes”. For example, TRIM20 lacks a catalytic RING domain but can still assemble active autophagy initiation complexes (Kimura et al., 2015). On the other hand, TRIM28 has been shown to enhance the PI3 kinase activity of Beclin 1 complexes by directly SUMOylating hVPS34, and TRIM50 attaches K63-linked poly-ubiquitin to Beclin 1 in an autophagy-activating manner (Yang et al., 2013; Fusco et al., 2018). TRIM32 has been reported to promote the activity of the ULK1 complex through the generation of unattached K63-linked poly-ubiquitin chains (Di Rienzo et al., 2019). Whether the enzymatic activity of other autophagy-regulating TRIMs is required for their actions in autophagy remains to be answered.

### TRIMs Control Autophagic Substrate Selectivity

One of the primary ways that autophagy can impact cellular health and physiology is through the degradative elimination of cytoplasmic contents. Once considered a bulk cellular recycling mechanism, the autophagy pathway is now known to selectively target certain substrates for degradation. This selective autophagy presents an opportunity for the potential deployment of autophagy-modulating therapies. While the wholesale induction or inhibition of autophagy may have deleterious side effects, the still-theoretical ability to activate or inhibit the autophagic degradation of a specific cancer-related target could be considerably safer since only some of autophagy's many physiological roles would be impacted.

The autophagy machinery's ability to selectively recognize substrates is based on proteins that act as autophagy receptors. These receptors are thought to act by bridging autophagic cargoes with mAtg8s associated with the nascent autophagosomal/phagophore membrane. Receptors can interact with cargos directly or indirectly through a protein “eat-me” tag; these tags are often ubiquitin-based (Kirkin et al., 2009). Receptors interact with mAtg8s *via* two different defined peptide sequences termed LC3-interacting regions (LIRs) (Birgisdottir et al., 2013) or ubiquitin interacting motif-like (UIM) (Marshall et al., 2019). The best recognized autophagy receptors are the sequestosome-like receptors (SLRs), which include the proteins p62/Sequestosome 1, NDP52, NBR1, Optineurin, and TAX1BP1. These proteins all include ubiquitin binding domains for substrate recognition and LIRs, and these domains have been shown to be important for these proteins to carry out the autophagic degradation of specific proteins, organelles, or intracellular pathogens. Autophagy receptors also have autophagy-regulatory roles by linking selective autophagy substrates with upstream autophagy regulators as exemplified by NDP52, which recruits the ULK1/FIP200 complex to depolarized mitochondria during mitophagy (Vargas et al., 2019). In addition to regulating the autophagy pathway, multiple TRIMs impact the autophagic targeting and degradation of select substrates by themselves acting as autophagy receptors or by modulating the actions of SLRs.

Most TRIM family members have N-terminal RING catalytic domains that act as E3 ubiquitin ligases. As such, it may be expected that TRIM-mediated ubiquitination of autophagy substrates leading to their recognition by ubiquitin binding receptors such as the SLRs would be a common mechanism of TRIM-mediated selective autophagy. However, to date this mechanism is not well-established; although there is an indication that TRIM21-mediated ubiquitination of the kinase IKKβ may facilitate IKKβ degradation by autophagy (Niida et al., 2010). On the contrary, TRIM14 and TRIM59 have been shown to prevent the ubiquitination and subsequent p62-mediated autophagic degradation of the DNA sensing enzyme cGAS (Chen M. et al., 2016) and PDCD10 (programmed cell death protein 10) (Tan P. et al., 2018). Interestingly, the TRIM59-mediated protection of PDCD10 from autophagy was shown to promote the survival and growth of breast cancer cells (Tan P. et al., 2018).

Instead of tagging autophagy substrates with ubiquitin “eat-me tags”, TRIMs appear to act as autophagy receptors that directly bind to their substrates (**Table 2**). This was originally demonstrated for TRIM5 (Mandell et al., 2014), a protein that is known to assemble into a lattice around incoming retroviral cores in a host- and viral-species specific manner (Ganser-Pornillos and Pornillos, 2019). TRIM5 was found to include two LIR motifs and to bind directly to mAtg8 proteins (Mandell et al., 2014; Keown et al., 2018). Rhesus TRIM5 promoted the autophagy-dependent degradation of HIV-1 viral components (Mandell et al., 2014), which can be recognized and bound by the TRIM5 SPRY domain (Stremlau et al., 2006). Binding to mAtg8s appears to be a feature of multiple TRIMs in addition to TRIM5 (Pizon et al., 2013; Mandell et al., 2014; Kimura et al., 2015; Kimura et al., 2016; Overå et al., 2019). This feature puts these mAtg8-binding TRIMs into a position where they can recruit their interacting partners to autophagosomes for degradation. Autophagic degradation of cancer-relevant targets is one possible mechanism explaining how autophagy may impact cancer progression (**Table 2**). Given the large size of the TRIM family, it is possible that TRIMs provide cells with a breadth of selective autophagy receptors.

**Table 2:** Substrates whose autophagic degradation is controlled by TRIMs. Top, in some cases, TRIMs promote the selective autophagic degradation of the listed substrates. In other cases (bottom), TRIMs ‘deselect’ potential autophagic substrates allowing them to accumulate in cells.

	Autophagic substrate	TRIMs involved	Cancer relevance
**TRIM-mediated selective autophagy**	Cleaved caspase-3	TRIM8 (Roy et al., 2018)	Cleaved (active) Caspase-3 is essential to apoptosis.
	AIM2 inflammasome	TRIM11 (Liu et al., 2016)	AIM2 inflammasome inhibits the development of colorectal cancer but promotes squamous cell carcinoma. AIM2 inflammasome triggers cell death and inflammation in response to DNA damage.
	Endoplasmic reticulum	TRIM13 (Ji et al., 2019)	Endoplasmic stress can enhance tumorigenesis, metastasis, and drug resistance. ER stress can also attenuate anti-cancer immunity.
	Aggregated proteins	TRIM16 (Jena et al., 2018), 50 (Fusco et al., 2012)	Misfolded proteins lead to aggregate formation. Cancer cells utilize the degradation of aggregates through autophagy to facilitate cell survival.
	Damaged lysosomes	TRIM16 (Chauhan et al., 2016)	Induced lysosomal damage has been considered as an approach to cancer chemotherapy.
	Midbody rings	TRIM17, 21, 76 (Mandell et al., 2016)	The midbody is the compacted remains of the cytokinesis machinery. Midbodies accumulate in cancer stem cells and have been linked to cancer invasiveness.
	NLRP3 inflammasome components	TRIM20 (Kimura et al., 2015)	NLRP3 inflammasome regulates the activation of pro-inflammatory cytokines that can have strong effects (protective and pathogenic are both reported) on cancer.
	IRF3	TRIM21 (Kimura et al., 2015)	IRF3 is a transcription factor that is activated in response to cellular pathogen detection. IRF3 inhibition slows gastric tumor growth.
	Active IKKβ	TRIM21 (Niida et al., 2010)	IKKβ de-represses NF-κB-based gene expression
	TRIF	TRIM32 (Yang et al., 2017)	TRIF is an adaptor protein that is important for Toll-like receptor signaling. TLR signaling has been linked to cancer progression.
**TRIM-mediated deselective autophagy**	TGFβ activated kinase 1 (TAK1) complex components	TRIM5 (Kehl et al., 2019)	TAK1 is a kinase that integrates signaling downstream of TGFβ and other cytokines and has been extensively linked to cancer. TAK1 inhibition has been considered in cancer therapy.
	Cyclic GMP-AMP synthase (cGAS)	TRIM14 (Chen M. et al., 2016)	cGAS is crucial for cytosolic DNA sensing. cGAS Acute activation of cGAS has been shown to lead to tumor regression in mice, whereas chronic cGAS activation may lead to inflammation-induced tumorogenesis.
	Intraflagellar transport 20 (IFT20)	TRIM17 (Mandell et al., 2016)	IFT20 is involved in ciliogenesis and microtubule-driven transport. Primary cilia are thought to inhibit cell growth and are lost in many cancers.
	Oral-facial-digital syndrome 1 (OFD1)	TRIM17 (Mandell et al., 2016)	OFD1 is an inhibitor of primary ciliogenesis. Primary cilia are thought to inhibit cell growth and are lost in many cancers.
	TRIM22	TRIM17 (Mandell et al., 2016)	TRIM22 expression is associated with poor prognosis in non-small cell lung cancer (Liu et al., 2017b).
	Programmed cell death protein 10 (PDCD10)	TRIM59 (Tan P. et al., 2018)	PDCD10 inhibits RhoA/ROCK signaling, thus promoting cancer cell survival and metastasis.

While ubiquitin tagging of substrates by TRIMs has not yet been definitively reported, several studies indicate that auto-ubiquitination of TRIMs when bound to their substrates is important for their actions as autophagy receptors. For instance, TRIM11 binds to the DNA sensor AIM2 following activation by cytoplasmic DNA. TRIM11 then auto-ubiquitinates at lysine 458. This modification is required for p62 recruitment and AIM2 degradation by autophagy (Liu et al., 2016). AIM2 has been suggested to play roles in several cancers, and the TRIM11-p62-autophagy axis attenuated AIM2 signaling. Auto-ubiquitination is required for the recently uncovered roles of TRIM13 in ER-phagy (the autophagic targeting of damaged endoplasmic reticulum) (Ji et al., 2019). Analogously, auto-ubiquitinated TRIM32 binds to the signaling adapter TRIF and acts as an autophagic “eat-me” signal that is detected by TAX1BP1 (Yang Q. et al., 2017). This raises the question as to how TRIM auto-ubiquitination is regulated. In the case of TRIM5, the spatial arrangement of TRIM5 dimers scaffolded on a retroviral core allows the RING domain of one TRIM5 molecule to poly-ubiquitylate the RING domain on another TRIM5 molecule (Fletcher et al., 2018). Thus, substrate recognition may be required for TRIM auto-ubiquitination and action as autophagy receptors. This is likely the case for TRIM17. Under normal conditions, TRIM17 assembles Beclin 1 with an inhibitory binding partner to inhibit autophagy. However, TRIM17-Beclin 1 complexes localized to midbody rings lack this inhibitory binding partner, and TRIM17 contributes to the autophagic elimination of midbody rings (Mandell et al., 2016). Whether and how TRIMs coordinate their substrate binding activities as selective autophagy receptors with their enzymatic activities and their actions as autophagy regulators (described above) remains an open question.

By definition, autophagy receptors are co-degraded with their targets in the autolysosome. So far, autophagic degradation has been demonstrated for at least TRIMs 5, 13, 16, 20, 21, 23, 27, 31, 32, 45, 49, 50, and 56 (Imam et al., 2016; Mandell et al., 2016; Ji et al., 2019; Overå et al., 2019), several of which have known connection to cancer (**Table 1**). This finding raises the autophagic degradation can also regulate the cancer-related activities of these TRIMs. Interestingly, this effect is seen with the oncogenic fusion protein PML-RARα (TRIM19), whose autophagic degradation is induced in following exposure to all-trans retinoic acid (Isakson et al., 2010; Wang et al., 2011), which is a standard treatment for acute promyelocytic leukemia (Wang and Chen, 2008).

### TRIMs Control the Activities of the Cancer-Related Autophagy Receptor and Signaling Platform p62/Sequestosome 1

The protein p62/Sequestosome 1 (p62) has multiple known roles in a variety of cancers (Moscat et al., 2016; Sanchez-Martin et al., 2019). The best known cellular function of p62 is as a selective autophagy receptor (Pankiv et al., 2007; Deretic, 2012). Separate from its actions in autophagy, p62 also plays a key role in a number of cellular signaling pathways that can profoundly affect cellular survival and growth (Sanz et al., 2000; Jin et al., 2009; Jain et al., 2010; Komatsu et al., 2010; Duran et al., 2011; Linares et al., 2013; Paul et al., 2014; Moscat et al., 2016; Goodall et al., 2016; Sanchez-Martin and Komatsu, 2018). Numerous studies have reported p62 over-expression in tumors and have associated elevated p62 expression with poor prognosis (Duran et al., 2008; Inui et al., 2013; Li et al., 2013; Adams et al., 2016; Saito et al., 2016; Umemura et al., 2016; Karras et al., 2019; Nguyen et al., 2019; Polonen et al., 2019). Furthermore, p62 has been shown to limit the efficacy of sorafenib treatment against liver cancer (Sun et al., 2016). Given its multifunctional nature, it is unsurprising that dysregulation of p62 in cancer cells can promote their growth by several mechanisms including through selective autophagy (Nguyen et al., 2019), activation of pro-survival signaling and gene expression (Duran et al., 2011; Linares et al., 2013; Umemura et al., 2016; Lam et al., 2017; Polonen et al., 2019), or stabilization of a set of pro-metastatic mRNAs (Karras et al., 2019). In contrast, p62 expression in non-transformed cells can reduce cancer progression (Valencia et al., 2014; Huang et al., 2018). In light of all of p62's possible pathogenic or protective effects in cancer, a key question is how p62 coordinates its various cellular activities and what factors govern its behavior.

The control of p62 action is emerging as a conserved feature of TRIM family members. As discussed above, TRIMs can regulate the levels of p62 indirectly through their actions as autophagy regulators. However, TRIMs employ additional mechanisms for affecting p62 abundance and activity. TRIMs 5,11,13, 17, 21, 22, 23, 32, 49, 50, 55, and 63 have been demonstrated to biochemically interact with p62 (Witt et al., 2008; O'Connor et al., 2010; Fusco et al., 2012; Tomar et al., 2012; Mandell et al., 2014; Kimura et al., 2015; Liu et al., 2016; Pan et al., 2016; Sparrer et al., 2017; Overå et al., 2019), and additional TRIMs such as TRIM19 colocalize with p62 in cellular structures (Clausen et al., 2010). A primary cellular function of p62 is to organize and sequester ubiquitylated proteins into cytoplasmic punctate structures termed p62 bodies which have liquid droplet-like properties (Zaffagnini et al., 2018) and that may function as platforms for p62-mediated signaling while also concentrating cellular wastes destined for autophagic degradation. At least 14 TRIMs have roles in regulating the formation and/or clearance of these structures, with TRIMs 5, 16, 17, 32, 50, 52, and 58 increasing their abundance in cells while TRIMs 14, 19, 21, 22, 25, 65, and 76 having the opposite effect (Mandell et al., 2016; Pan et al., 2016; Jena et al., 2018; Kehl et al., 2019; Overå et al., 2019). The relevance of TRIM-regulated p62 localization to cancer is illustrated by TRIM21 and TRIM16, which act in opposing manners on p62 condensation into cytoplasmic bodies and on activation of the transcription factor Nrf2.

As a master transcriptional regulator of antioxidant stress resistance genes, Nrf2 is a prominent actor in cancers (Kitamura and Motohashi, 2018). Under homeostatic conditions, Nrf2 is targeted for proteasomal degradation through its interaction with Keap1. In response to oxidative stress, p62 binds to Keap1, leading to Keap1's sequestration and eventual autophagic degradation. This liberates Nrf2 from Keap1, allowing Nrf2 to enter the nucleus and to activate its target genes (Komatsu et al., 2010; Taguchi et al., 2012; Ichimura et al., 2013). TRIM21 directly catalyzes the K63-linked poly-ubiquitylation of p62 at lysine 7 in the p62 PB1 domain, a region required for p62 dimerization and cytoplasmic body formation. Consequently, K7-ubiquitylated p62 loses its ability to sequester Keap1, establishing TRIM21 as a negative regulator of Nrf2-directed cytoprotective antioxidant responses and providing a possible mechanism explaining the observation that reduced TRIM21 expression is associated with poor prognosis in hepatocellular carcinoma and B cell lymphoma (Brauner et al., 2015; Ding et al., 2015). In contrast, TRIM16 is required for the formation of p62 bodies in response to proteotoxic or oxidative stress, and its expression decouples Nrf2 from its inhibitor Keap1 (Jena et al., 2018). Knockout of TRIM16 reduces cellular survival and growth under stress conditions *in vitro* and in a mouse xenograft tumor model (Jin et al., 2009). While the precise molecular mechanism of TRIM16 action on the p62-Nrf2 system has not been completely defined, TRIM16 expression is associated with increased p62 phosphorylation (Ichimura et al., 2013), suggesting that TRIM16 may be involved in the activation of a kinase that controls p62 action. A likely candidate for this role is TAK1, a kinase activated downstream of TRIM5 (Pertel et al., 2011) and other TRIMs (Versteeg Gijs et al., 2013), that phosphorylates p62 (Hashimoto et al., 2016; Kehl et al., 2019) and is required for p62 body formation in response to multiple cellular stresses (Kehl et al., 2019). How other TRIMs affect p62 localization and Nrf2 activation, and what role(s) these activities play in cancer has not been fully elucidated.

### Possible Approaches to Drugging TRIMs in Cancer Therapy

Can the connections between TRIMs and autophagy be leveraged for the therapeutic benefit of cancer patients? While this concept has not yet been tested, there is reason to believe that TRIMs could be druggable targets. TRIMs are a heterogeneous group of proteins organized into subclasses that possess a defining cluster of domains (e.g. RING, B-box, coiled-coil, FN3, SPRY, bromobox/bromodomain, etc.) (Gushchina et al., 2018). One approach to drug design would be to target the activity of specific domains critical to TRIM function in cancer. In fact, efforts to identify inhibitors of TRIM bromodomains are underway, with the bromodomains present in TRIM24, 28, and 33 being of particular interest to cancer therapy. These three TRIMs are transcriptional modulators associated with multiple cancers (**Table 1**). Bromodomains are involved in the recognition of acetylated lysines on histones and can recruit chromatin remodeling enzymes, resulting in transcriptional activation or repression including of autophagy-related genes (Sakamaki et al., 2017). Small-molecule bromodomain inhibitors have been identified that display robust target specificity, including against TRIM24 (Zhan et al., 2015; Bennett et al., 2016; Romero et al., 2016). Such agents may serve as useful epigenetic based anti-cancer therapies. As discussed above, bromodomain-containing TRIM28 has multiple connections to autophagy, and TRIM33 was among TRIM “hits” as regulators of autophagy. The role of the bromodomain of these TRIMs in their autophagic function(s) has not yet been tested, but one could imagine that bromodomain-targeted therapies could also impact autophagy directed by bromodomain-containing TRIMs.

The RING domain found in most TRIMs may also present an opportunity for therapeutic targeting. Most TRIM RINGs possess E3 ubiquitin ligase activity that is often crucial to TRIM functionality. While inhibitors specific to TRIM RING domains have not yet been reported, the fact that small molecule inhibitors of the RING domains from other protein families exist (Bulatov et al., 2018) suggests that TRIM RING inhibition may be feasible. The search for E3 ligase inhibitors in general has been sparked by the desire to identify molecules that could modulate ubiquitination-dependent proteasomal degradation of selected proteins. This process has been targeted therapeutically using drugs such as bortezomib, a 26S proteasome inhibitor, which is used in the treatment of cancers including multiple myeloma. Bortezomib's utility in cancer therapy is limited by toxicity, possibly resulting from the general, non-specific nature of proteasomal inhibition. However, because E3 ubiquitin ligases have some level of specificity in their action, their pharmacological targeting may provide greater therapeutic utility. A prime example of this approach that is currently under investigation is to target interactions between TRIMs and p53, a notable tumor suppressor (Valletti et al., 2019). Inhibiting the E3 ligase activity of these TRIMs to improve p53 stability may represent a selective therapeutic target for cancer. Inhibition of the E3 ligase activity of TRIMs would also impact their ubiquitination-dependent but proteasome independent activities, including those in autophagy regulation and in autophagic substrate selection.

A third possible modality for TRIM-directed cancer therapy would be to interfere with or enhance the interactions between a TRIM and its cancer-relevant binding partners. Depending on the TRIM and its mechanism of action in cancer, these interacting partners could include autophagy factors (e.g. Beclin 1, p62, mAtg8s). Alternatively, compounds capable of disrupting the oligomerization of cancer-relevant TRIMs would be expected to block their action, since the higher-order assembly of TRIMs is thought to be essential for their function. In total, there is strong support for targeting TRIMs in an effort to develop effective therapies for a wide-array of cancers.

## Concluding Remarks

TRIM proteins are positioned as hubs connecting cellular signaling, metabolism, and autophagy. As such, it is unsurprising that so many of them have prominent roles in cancer. To date, no efforts to effectively target TRIMs as a strategy to therapeutically modulate autophagy in cancer have been reported. However, the strong connections between TRIMs and a variety of diseases in addition to cancer suggest that TRIM targeting may hold promise pending future mechanistic studies. These studies could be initiated by two complementary approaches. First, the requirement for autophagy should be investigated in cancers showing TRIM dysregulation. Second, TRIMs should be assessed for whether they promote the survival of autophagy-addicted cancers. Either approach might identify cancers in which TRIM-directed autophagy plays a significant role in tumor survival and/or resistance to chemotherapeutic agents. This could justify further cellular, biochemical, and structural studies aimed at identifying TRIM structures or activities to target in the development of more effective cancer therapies.

## Author Contributions

MM and BS wrote the sections on autophagy regulation by TRIMs. TT wrote the sections on the connections between TRIMs and cancer and on TRIMs as therapeutic targets. Other sections were written collaboratively by all three authors.

## Funding

MM is supported by P20GM121176 and R21AI131964 from NIGMS and NIAID, respectively.

## Conflict of Interest

The authors declare that the research was conducted in the absence of any commercial or financial relationships that could be construed as a potential conflict of interest.
